# Supplementary material for: The Cycle to Respectful Care: A Qualitative Approach to the Creation of an Actionable Framework to Address Maternal Outcome Disparities
Source: Int J Environ Res Public Health. 2021 May 6;18(9):4933. doi: 10.3390/ijerph18094933 (PMC8141109; doi:10.3390/ijerph18094933)
Supplement: Supplementary file 1 [file ijerph-18-04933-s001.zip › ijerph-1165420.pdf]

PREM FACILITATOR GUIDE

Facilitator Opening Statement of Purpose and Expectation:
We encourage you to share information about a range of experiences and emotions from satisfying and beautiful births to frustrating and challenging births. We want to hear it all. While many of us can relate to each other's experiences, we want to open the space for everyone to share their full story. Throughout your story, we will listen and may also ask you, during breaks or pauses in your story, to ask you "name the thing" that is causing you to feel so much emotion and passion about your story. For example, if you are talking about an interaction with hospital staff, I may ask you to clarify what you felt and "name it" as "respect" or "disrespect", "being heard or being ignored." First, we will begin with a grounding exercise to center us in the power and wisdom of language and creative expressions. Although we invited you here so we can learn more about your birth experiences, we want to emphasize that you have permission to skip questions. You can simply say PASS when you don't want to participate, you can also choose silence. Your participation is voluntary which means that at any moment you can choose if, when, and how you want to share or not share and/or you want to participate or not participate.

Readiness and Responsiveness to Birth

1. **Did you do anything to prepare for birth?**
   - What things did you find to most helpful or not helpful at all?
2. **From whom or where did you seek help or information, when you had questions or concerns about birth and lactation?**
   - How satisfied were you the support and information you received?
   - What about this person or place made it easy to ask questions or share your concerns? What about this person or place made it difficult to ask questions or share your concerns?
   - What made it easier or harder to get support and information you needed about birth and lactation?

3. **What types of birth options did you learn about?**
   - How did you make your birth preference(s) known to the hospital staff?
   - Do you feel your voice was heard?
     - [YES] What did you do to make your voice heard?
     - [NO] Tell me more about that.
   - What things made it easier or harder to be noticed?
   - What things made it easier or harder to be heard?
   - What things made it easier or harder to get what you wanted for your birth?
   - Probing: Think of the times you went to the hospital to register, to go to lab, get your ultrasound, take a tour of the Birthing Unit, or attend prenatal or childbirth education classes

Birth Expectations and Experiences

4. **In this moment, what do you want your hospital to know about your experience with your labor, birth, and recovery?**

- How did your birth experience in the hospital compare to what you imagined or dreamed your experience would be?
- Who did you want to be in the birth space with you? Were they able to be in the birth space with as you had imagined? Tell me more about that.

5. **Did you get all the care you needed during your birth and afterwards?**
   - Do you believe you received too much care?
   - Do you believe you received too little care?
   - Were you screened for postpartum depression or mental/emotional health?
   - Did you ever feel ignored, forgotten, misunderstood, or neglected during birth or afterwards? Tell us more about that.
   - What was missing from your birth care?
   - What were some of things that prevent you from or got in the way of your getting the care you needed during your birth? This could be anything from having insurance, the type of insurance, childcare for current children, the cost of hospital care, transportation, skin color, body size and shape, attitude, tone of voice, word choice, being alone/no support, lack of lack of information to make different decisions or being treated poorly by providers.
   - What would you do differently during your birth?
   - If you felt traumatized by your birth experience, what was the interaction with the system and/or providers that caused it?
   - If you felt traumatized during your birth experience, how was it handled by the system and/or providers?

6. **What type of birth care is missing for Black women?**
   - This is your chance to change and influence the ways in which Black women experience hospital births in California. What would you tell anyone who was a part of your birth experience to do differently? For example. what would you tell your doctor to do differently? What would you tell the nurses to do differently? Or the midwife, doula, receptionist, Uber driver?

<u>Birth Supports and Resources</u>

7. **What types of supports and resources did you have or use during and after childbirth, such as counseling, doula support, lactation consultant, social services, or peer support group?**
   - What types of support and resources were missing or found to be inadequate?
8. **Who was someone throughout your pregnancy and childbirth experience who made a difference?**
9. **What were some of the things that concerned or worried you about your treatment in the hospital? In what ways were these worries consistent with your actual experience?**
   - What were some of the things that you felt good or confident about with your treatment in the hospital? In what ways were these things consistent with your actual experience?

10. **What were some of reasons or things that helped you to get care you wanted and needed during your birth?**
    - This could be anything from having a midwife, having a doula, having a strong birth support team, having insurance, type of insurance, childcare for current children the cost of care, transportation, skin color, body size and shape, attitude, tone of voice, word choice, being alone/no support, having information to make informed decisions, being treated respectfully by providers, being listened to, and having providers respond to my questions and concerns in a timely and appropriate manner, my own power and voice.

Birth Priorities and Autonomy

11. **Tell us what you know about your community and the ways in which it influences your ability to have the birth you desire.**
    - What knowledge and/or access to midwives, Black/POC midwives, doulas, Black/POC doulas, home births options, birthing center options, water births in hospital settings, Baby Friendly Hospitals, Lactation Friendly Hospitals, lactation educators and consultants, childbirth education classes for Black birthing communities, VBAC Friendly Obstetricians and Hospitals, Reproductive Justice Informed Midwives and Obstetricians do you have in your community?

12. **Tell us what you know about your local hospital and how they influenced your ability to have the birth you desire.**

13. **Was there anything going on with the health care team or the hospital environment that stressed you out or caused you anxiety during your birth?**
